# Epigenetic Signature: A New Player as Predictor of Clinically Significant Prostate Cancer (PCa) in Patients on Active Surveillance (AS)

**DOI:** 10.3390/ijms18061146

**Published:** 2017-05-27

**Authors:** Matteo Ferro, Paola Ungaro, Amelia Cimmino, Giuseppe Lucarelli, Gian Maria Busetto, Francesco Cantiello, Rocco Damiano, Daniela Terracciano

**Affiliations:** 1 Urologic Surgery Unit, European Institute of Oncology, 20141 Milan, Italy; matteo.ferro@ieo.it; 2 Institute of Experimental Endocrinology and Oncology (IEOS-CNR) “G. Salvatore”, Via Sergio Pansini, 5, 80131 Naples, Italy; 3 Institute of Genetics and Biophysics “A. Buzzati Traverso”, National Research Council (CNR), Via Pietro Castellino 111, 80131 Naples, Italy; 4 Department of Emergency and Organ Transplantation-Urology, Andrology and Kidney Transplantation Unit, University of Bari, 70124 Bari, Italy; giuseppe.lucarelli@inwind.it; 5 Department of Gynecological-Obstetrics Sciences and Urological Sciences, Sapienza Rome University Policlinico Umberto I, 00161 Rome, Italy; gianmaria.busetto@uniroma1.it; 6 Department of Urology, Magna Graecia University of Catanzaro, 88100 Catanzaro, Italy; cantiello@unicz.it (F.C.); damiano@unicz.it (R.D.); 7 Department of Translational Medical Sciences, University of Naples Federico II, Via Sergio Pansini, 5, 80131 Naples, Italy

**Keywords:** active surveillance, prostate cancer, epigenetic biomarkers

## Abstract

Widespread prostate-specific antigen (PSA) testing notably increased the number of prostate cancer (PCa) diagnoses. However, about 30% of these patients have low-risk tumors that are not lethal and remain asymptomatic during their lifetime. Overtreatment of such patients may reduce quality of life and increase healthcare costs. Active surveillance (AS) has become an accepted alternative to immediate treatment in selected men with low-risk PCa. Despite much progress in recent years toward identifying the best candidates for AS in recent years, the greatest risk remains the possibility of misclassification of the cancer or missing a high-risk cancer. This is particularly worrisome in men with a life expectancy of greater than 10–15 years. The Prostate Cancer Research International Active Surveillance (PRIAS) study showed that, in addition to age and PSA at diagnosis, both PSA density (PSA-D) and the number of positive cores at diagnosis (two compared with one) are the strongest predictors for reclassification biopsy or switching to deferred treatment. However, there is still no consensus upon guidelines for placing patients on AS. Each institution has its own protocol for AS that is based on PRIAS criteria. Many different variables have been proposed as tools to enrol patients in AS: PSA-D, the percentage of freePSA, and the extent of cancer on biopsy (number of positive cores or percentage of core involvement). More recently, the Prostate Health Index (PHI), the 4 Kallikrein (4K) score, and other patient factors, such as age, race, and family history, have been investigated as tools able to predict clinically significant PCa. Recently, some reports suggested that epigenetic mapping differs significantly between cancer patients and healthy subjects. These findings indicated as future prospect the use of epigenetic markers to identify PCa patients with low-grade disease, who are likely candidates for AS. This review explores literature data about the potential of epigenetic markers as predictors of clinically significant disease.

## 1. Introduction

AS has recently become an accepted alternative for patients with low-risk prostate cancer (PCa)-related mortality, allowing for delayed curative intervention if there is reclassification of cancer risk or evidence of disease progression [1]. It has been widely accepted that pre-treatment prostate-specific antigen (PSA) below 10 ng/mL, a biopsy Gleason score of 6 or less, and clinical stage T1c or T2a identify low-risk PCa [2]. Moreover, Klotz et al. [3] recently suggested that patients with a Gleason score of 3 + 4 PCa may be selected for active surveillance (AS), since most of these patients are at a low risk of progression. Risk factors for reclassification and progression have still not been adequately characterized. Low-risk PCa patients were monitored through PSA levels, clinical examination, and repeated prostate biopsies. Changes in biopsy results suggest a need for intervention. Widespread AS use has been prevented because under-sampling prostate biopsies may result in occult high-grade cancer. Moreover, biopsies are invasive tests, not free from side effects such as bleeding and infection risks, suggesting a need to avoid unnecessary repeated prostate biopsies in AS regimens. Several approaches to selecting patients for AS have been proposed. One of these includes the addition of biomarkers to currently used clinical and demographical variables [4]. Biomarker levels can ideally be obtained non-invasively, allowing for controlled follow-up of patients in AS in order to avoid overtreatment and the consequent impairment of quality of life.

In this review, we focused our attention on the potential use of epigenetic mapping as prognostic factors in the clinical management of PCa patients.

### 1.1. Biomarkers and Active Surveillance: Current Status

PCa patients are classified at diagnostic biopsy as very low-risk when they meet the following criteria: clinical stage T1c disease, PSA-D less than 0.15 ng/mL, a Gleason score ≤6, two or fewer biopsy cores with cancer, and a maximum of 50% involvement of any core with cancer [5]. Thaxton et al. showed that the number of patients eligible for AS, who have fatal disease at radical prostatectomy (RP), depends upon the criteria used for AS selection [6]. However, the optimal patient selection and follow-up protocol is still a matter of debate. Prostate biopsy risks, such as bleeding and infections, highlight the need for non-invasive tools for the selection and follow-up of AS patients [7]. The Prostate Health Index (PHI) may play a role in monitoring men under active surveillance (AS) [8,9]. Tosoian et al. [10] found an association between baseline and longitudinal PHI, but not PCA3 [11] values and reclassification during active surveillance. Sottile et al. [12] demonstrated significantly higher p2PSA and PHI levels in men with metastatic disease as compared to those without clinical metastasis. Collectively, PHI and/or PCA3 improve the selection of eligible patients for AS and decision curve analysis demonstrated that PHI outperforms PCA3 [13]. A Canadian report indicated that neutrophil-to-lymphocyte ratio (NLR) is a less expensive and more easily accessible test able to predict Gleason score upgrading and biochemical recurrence in patients with low-risk PCa eligible for AS [14]. Recently, Ferro et al. [15] found a significant association between low serum testosterone levels and upgrading, upstaging, and unfavorable disease, suggesting a new cheap parameter useful to identify low-risk patients eligible for AS. A recently published preliminary study [16] showed that the presence of primary circulating prostate cells is associated with aggressive disease, suggesting that these patients are not eligible for active surveillance. Several authors have shown that, besides the PHI index, the PCA3 score can improve the Epstein and PRIAS protocol’s ability to predict insignificant PCa in subjects eligible for AS [17,18]. Lin et al. found that [19] the combination of urine TMPRSS2-ERG and PCA3 were associated with aggressive cancer in men with low-risk PCa on AS. Berg et al. found a significant association between tissue ERG expression and progression during AS [20]. Other authors have recently shown that Ki67 and DLX2, two cancer cell proliferation markers, are predictive of increased metastasis risk and may aid patient selection for AS [21]. It has been repeatedly demonstrated [22,23,24] that PTEN loss is uncommon in clinically localized PCa, suggesting the potential use of this histopathological biomarker as a predictor of unfavorable prognosis in patients on AS.

Tissue-based prognostic panels as OncotypeDX1 and Prolaris1 have been validated for routine clinical use on men with low-risk PCa. The first is a quantitative RT-PCR assay performed using biopsy samples. This test measures the expression of several genes involved in four different pathways to calculate the Genomic Prostate Score (GPS), which is predictive of aggressive disease in low- and intermediate-risk PCa patients [25]. The Prolaris 1 test provides a proliferative index, called CCP (cell cycle progression) score, on the basis of the expression of 31 cell cycle progression and 15 housekeeping genes [26]. In a multicentric study, the biopsy CCP score from low-risk patients was associated with aggressiveness at radical prostatectomy [27].

### 1.2. Epigenetic Biomarkers and PCa Prognosis: Future Challenge

The term “epigenetics” defined heritable changes in gene expression that are independent from those occurring in the genome. The epigenetic mechanisms contribute to gene regulation throughout the whole life course of an organism, by changing chromatin architecture and/or access by transcription factors. They include different processes, such as DNA methylation, histone modifications, and post-transcriptional gene regulation by non-coding RNAs. Together, they regulate gene expression by changing chromatin organization and DNA accessibility.

The most well studied epigenetic modification in human diseases is DNA methylation. It involves an enzymatic process mediated by DNA methyltransferases (DNMTs) that catalyze the addition of a methyl group, using S-adenosyl methionine (SAM) as the methyl supplier, to the 5-carbon of the cytosine within CpG dinucleotides to form 5-methylcytosine. CpG are normally methylated when dispersed in the genome or in DNA repetitive region, but remain unmethylated to enable gene expression when they are clustered as a CpG island at the 5’ ends of many genes [28]. The conventional view is that DNMT1 is responsible for the maintenance of tissue-specific methylation patterns during cell replication, while DNMT3A and DNMT3B catalyze the addition of methyl groups de novo during embryogenesis [29,30]. In tumorigenesis, DNA methylation and demethylation are associated with silencing tumor suppressor genes and activating oncogenes, respectively [31].

Histone post-translational modifications (PTMs) include acetylation, biotinylation, methylation, phosphorylation, ubiquitination, SUMOylation, ADP (adenosine diphosphate) ribosylation, proline isomerization, citrulination, butyrylation, propionylation, and glycosylation, which are known as “the histone code” and strongly contribute to the control of gene expression [32].

Such modifications alter the affinity of the histone tails to the DNA and change the conformation of chromatin structure, resulting in transcriptional genes activation or repression. For instance, di- and trimethylation and poor acetylation of lysine 9 residue on histone H3 are associated with the silencing of gene expression. By contrast, the acetylation of histones H3 and H4, together with the methylation of lysine 4 residue on histone H3, results in gene expression. In general, acetylation promotes transcriptional activity and is catalyzed by histone acetyltransferases (HAT). Conversely, histone deacetylases (HDACs) remove acetyl groups leading to a silent chromatin state. Depending on the specific amino acid residues modified and the number of methyl groups added, histone methylation may be associated with the activation or repression of transcription. In general, histone methyltransferases (HMTs) and histone demethylases (HDMs) catalyze the addition or the removal of methyl groups from histone proteins, respectively [33].

There is good evidence that another epigenetic modification, known as noncoding RNAs (ncRNAs), can influence gene expression [34].

The best-characterized class of non-coding RNAs is represented by microRNA (miRNAs), which are single-stranded RNAs, about 19–24 nucleotides in length. They regulate gene expression through the binding to mRNAs, resulting in degradation or translational inhibition [35]. It was estimated that at least 30% of human genes are regulated by miRNA. In metazoans, each mRNA can be combined with multiple miRNAs and each miRNA regulates multiple mRNAs. It is noteworthy that miRNAs regulate a large spectrum of biological processes and play an important role in tumorigenesis, activating oncogenes or restraining tumor suppressor genes [36,37].

**Figure 1:**
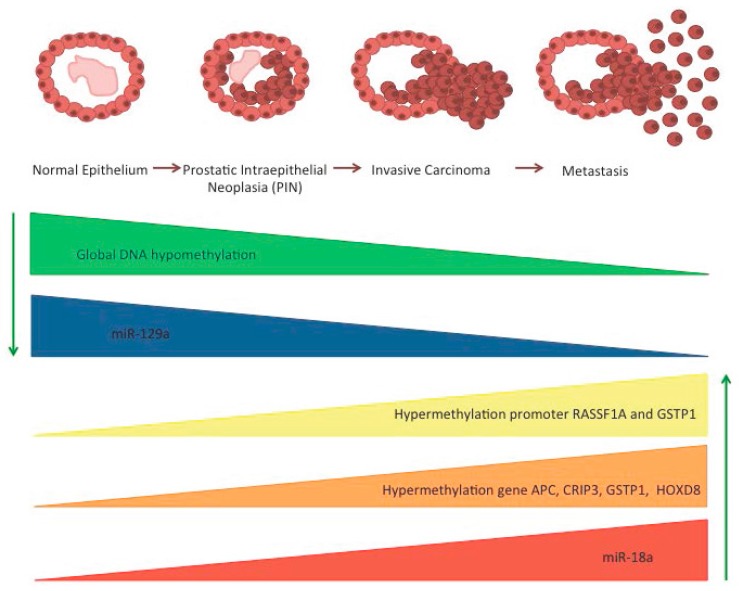
Potential epigenetic biomarkers in patients on AS. The figure shows the epigenetic modifications that have been tested as biomarkers. Green arrows represent down- and up-regulation of epigenetic biomarkers. The aberrant epigenetic changes have been described and cited in the text.

Development and progression of PCa are usually associated with global DNA hypomethylation with a lower overall content of 5-methylcytosine (m5C) found in metastatic tissue [38]. The global DNA hypomethylation in PCa causes a loss of IGF2 imprinting (with expression of both parental alleles) both in cancerous and in distant areas within the peripheral zone, indicating that the epigenetic defect in histologically normal tissue might be employed to identify PCa in patients [39] (Figure 1). Conversely, promoter hypermethylation is widespread during neoplastic transformation of prostate cells; indeed, this is one of the first aberrations, seen early in pre-invasive lesions, and appears to be clonally maintained during metastatic progression of PCa [40]. Thus far, several genes, including tumor suppressor genes, have been described as de novo methylated in morphologically normal prostate tissue and in pre-invasive lesions, such as PIN (Prostatic Intraepithelial Neoplasia), and persisting during prostate carcinogenesis [40]. As an example, the relative frequency of methylation of Ras association domain family protein 1, isoform A (*RASSF1A*) promoter was higher in more aggressive tumors compared to less malignant tumors [41,42]. Aberrant promoter methylation was also found in different genes involved in important molecular pathways of carcinogenesis, such as DNA repair/protection, cell cycle regulation, and signal transduction. As an example, the frequency of methylation at the promoter region of Glutathion S-transferase Pi 1 (*GSTP1*), a gene involved in DNA repair, was found elevated not only in more than 90% of PCa cases, but also in over 50% of PCa precursor lesions, confirming that this is an early event in prostate carcinogenesis [42,43,44]. Further supporting the relevance of DNA methylation in PCa progression, different authors have related CpG methylation patterns to clinical outcomes and revealed that methylation of certain loci (e.g., *AOX1* and *RARB*) predicted disease progression [45,46].

Histone modifications were also found to relate to the pathogenesis of prostate cancer and regulation of cancer cell proliferation. Ellinger and co-authors analyzed H3K4 methylation in patients with advanced PCa and found that this epigenetic modification was a significant predictor of PSA recurrence following radical prostatectomy. Moreover, they also found that H3K4me1, H3K4me2, and H3K4me3 levels were significantly increased in hormone-refractory prostate cancer (HPRC) [47]. One of the most studied epigenetic enzymes in PCa is the histone methyltransferase EZH2 responsible for H3K27 trimethylation. Its overexpression, particularly found in mCRPC [48], correlates with promoter hypermethylation and repression of some tumor suppressor genes [48,49]. Other epigenetic enzymes, such as SET9, SMYD3, JHDM2A, JMJD2C, and LSD1, have been demonstrated to play a role in prostate carcinogenesis [50,51,52,53,54]. LSD1, whose activity is associated to both transcriptional activation or repression, was associated with aggressive CRPC and a high risk of disease relapse [55,56]. Strong expression of all HDAcs was accompanied by enhanced tumor cell proliferation. High rates of HDAC1 and HDAC2 expression were significantly associated with tumor dedifferentiation, and HDAC2 expression is associated with shorter PSA relapse time after radical prostatectomy [57,58]. Moreover, AR transcriptional activity is regulated by HAT or HDAC activities, i.e., acetylation facilitates its binding to target DNA sequences, while HDAC1 and HDAC2 abrogate its activity [59]. These data confirm the role of HATs and HDACs in influencing the acetylation status of non-histone proteins. Additionally, the NAD-dependent deacetylase sirtuin-1 (SIRT1) is involved in PCa where its downregulation leads to the upregulation of different oncogenes as a consequence of H2A.Z overexpression [60].

Important biomarkers for PCa have been identified in microRNAs. Recently, Al-Kafaji et al. demonstrated that miR-18a expression increased in peripheral blood of patients with prostate cancer, indicating miR-18a as a potential noninvasive biomarker for prostate cancer tissue [61] (Figure 1). On the contrary, miRNA-129 was found downregulated in prostate cancer. Thus, its overexpression could prevent prostate cancer growth by developing tumor suppressive functions [62].

Zhao et al. [63] recently reported that a 4-gene methylation classifier panel (*APC*, *CRIP3*, *GSTP1*, and *HOXD8*) was able to predict patient reclassification on AS.

Epigenetic alterations are commonly found in PCa and play a role in carcinogenesis and tumor spreading. Moreover, new technologies, such as next-generation sequencing (NGS), have been implemented, allowing us both to expand our knowledge on prostate tumorigenesis and to obtain new epigenetic biomarkers useful to PCa patient clinical management.

Collectively, no single epigenetic biomarker has been identified as a marker of aggressive phenotypes.

However, several studies have identified potentially useful epigenetic biomarkers in PCa (Table 1) mainly in tissue samples.

The most studied epigenetic alteration is DNA methylation. Consequently, it is conceivable that methylation markers will be the first that will be translated into clinical practice for the management of PCa patients.

DNA methylation alterations, measured in cell-free circulating and urinary tumor DNA, can potentially be used as PCa biomarkers. A large number of specific DNA methylation alterations are cancer-specific and not detectable in unaffected subjects. Examples of such alterations may be represented by CpG island methylation in the regulatory regions of *GSTP1, APC, PTGS2, RASSF1A*, and *RARB* [64].

*GSTP1*, *RARB*, and *RASSF1* DNA promoter methylation has been widely investigated in body fluids as a non-invasive biomarker for the early diagnosis of PCa [65,66]. Other authors [67,68,69] have suggested that urine cell-free DNA could represent a non-invasive and inexpensive biomarker for assessing specific promoter region methylation.

Chromatin remodeling and non-coding RNA regulation represent an expanding research field.

Collectively, based on literature data and on the improvement of new technologies such as next-generation sequencing (NGS), epigenetic signatures seem to be promising tools for stratifying PCa patients for progression risk. Nevertheless, some obstacles may contribute to the lack of translating such biomarkers for PCa in clinical practice. In particular, an important role was played by the limitation of available methods for analysis, the consistency of experimental design to validate the biomarkers, and the relevance of the epigenetic alteration in prostate carcinogenesis. Moreover, it should be taken into account that epigenetic modifications are affected by aging and prostate cancer is an age-related disease.

Further studies on larger population will define the clinical benefit of epigenetic markers in body fluids.

## 2. Conclusions

Follow-up of patients on AS can prevent overtreatment and the related impairment of quality of life. Diagnosis of true low-risk PCa is essential to address this therapeutic strategy. As recently reported by Klotz [70], AS is harmless in the medium to long term with a very low cancer-specific mortality of 10–15 years. Many studies have been focused on tools able to provide further improvement of the safety of this conservative therapeutic option.

Several serum and urine biomarkers, including the PHI, the 4K score, and urinary TMPRSS2-ERG or PCA3 mRNA, have been evaluated in men on AS. However, the association with tumor aggressiveness and thus prognostic value remains controversial [71]. Consequently, new players have to be considered to predict cancer progression in an AS regimen.

In this scenario, it could be advantageous to implement epigenetic signature identification of clinically significant PCa in the setting of active surveillance. Such studies represent an urgent need to identify indolent cancer and avoid overtreatment. DNA methylation, histone modifications, and noncoding RNA could potentially provide new tools for prognosis of prostate cancer, affecting clinical management of patients. In particular, since these biomarkers lack specificity, further studies are needed to ascertain if a panel of multiple epigenetic targets may be helpful in planning AS strategies.

## Figures and Tables

**Table 1:** Overview of prostate cancer epigenetic biomarkers.

Biomarker	Type of Epigenetic Modification	Sample	References
IGF2	DNA hypo- and hyper-methylation	Tissue	[38,39,40]
RSSF1A	DNA hypermethylation	Tissue	[41,42]
GSTP1	DNA hypermethylation	Tissue	[42,43,44]
AOX1	DNA hypermethylation	Tissue	[45,46]
RARB	DNA hypermethylation	Tissue	[45,46]
EZH2	Increased H3K27 trimethylation	Tissue	[48]
SET9, SMYD3, JHDM2A, JMJD2C, LSD1	Histone modifications	Tissue	[50,51,52,53,54]
HAT	Variation in histone acetylation	Tissue	[59]
HDAC1, HDAC2	Histone deacetylation	Tissue	[59]
SIRT1	Downregulation	Tissue	[60]
miR-18a	Overexpression	Peripheral blood	[62]
miRNA-129	Downregulation	Peripheral blood	[62]
APC, CRIP3, GSTP1, HOXD8	DNA hypermethylation	Urinary	[63]
